# Neuroimaging in the diagnosis and treatment of cerebral toxoplasmosis in children with severe **β**-thalassemia after allo-HSCT

**DOI:** 10.17305/bb.2024.10708

**Published:** 2024-07-17

**Authors:** Meiai Liao, Guangrui Lai, Meiru Bu, Meiqing Wu, Muliang Jiang, Bihong T Chen

**Affiliations:** 1 Department of Radiology, The First Affiliated Hospital of Guangxi Medical University, Nanning, Guangxi, China; 2 Department of Hematology, The First Affiliated Hospital of Guangxi Medical University, Nanning, Guangxi, China; 3 Department of Diagnostic Radiology, City of Hope Comprehensive Cancer Center, Duarte, USA

**Keywords:** Cerebral toxoplasmosis, β-thalassemia major (β-TM), allogeneic hematopoietic stem cell transplantation (allo-HSCT), magnetic resonance imaging (MRI), fluorodeoxyglucose positron emission tomography/computed tomography, metagenomic next generation sequencing (mNGS)

## Abstract

Children with severe **β**-thalassemia major (**β**-TM) are at high risk of developing toxoplasmosis after allogeneic hematopoietic stem cell transplantation (allo-HSCT). The aim of this study was to identify the neuroimaging findings of cerebral toxoplasmosis in pediatric patient with **β**-TM for early diagnosis and treatment of cerebral toxoplasmosis. We performed a retrospective assessment of clinical and neuroimaging data of children with severe **β**-TM who had cerebral toxoplasmosis after allo-HSCT. Additionally, we reviewed and summarized the literature on cerebral toxoplasmosis in patients with other underlying conditions. This case series identified three children who had severe **β**-TM and had subsequent cerebral toxoplasmosis after allo-HSCT. In addition, we identified 23 patients from literature who had toxoplasmosis and had underlying conditions other than **β**-TM. We found that the most common clinical symptom among the patients from our series and the patients from literature was fever upon presentation. We identified the typical neuroimaging findings including brain lesions with ring enhancement and eccentric/central nuclear target-like enhancement, which should facilitate early diagnosis and treatment of cerebral toxoplasmosis.

## Introduction

Toxoplasma gondii (T. gondii) is an intracellular parasite that has affected people worldwide, with approximately 35.7 million people affected globally [1, 2]. The incidence of toxoplasmosis after hematopoietic stem cell transplantation (HSCT) ranges from 0.4% to 8.0% [3]. Humans can be infected with T. gondii through blood transfusions, organ transplantation, or HSCT [4]. Primary infection with Toxoplasma gondii typically does not cause symptoms in immunocompetent people and is usually identified through serological testing for anti-toxoplasma antibodies. However, in immunocompromised patients, such as those with acquired immunodeficiency syndrome (AIDS) or individuals undergoing allogeneic HSCT (allo-HSCT), toxoplasmosis often leads to life-threatening opportunistic infections and complications [5], with the central nervous system being the most affected site [6, 7].

The diagnosis of cerebral toxoplasmosis depends on the patient’s history of exposure, clinical symptoms and/or signs, characteristic neuroimaging findings, positivity for toxoplasma antibodies in cerebrospinal fluid (CSF) and/or serum, positivity for toxoplasma DNA by polymerase chain reaction (PCR) in blood and/or CSF, and pathogen detection in brain tissue and/or CSF [8]. However, cerebral toxoplasmosis can be difficult to diagnose due to the absence of typical clinical features and imaging findings in most patients, which leads to misdiagnosis and adverse outcome. Currently, metagenomic next generation sequencing (mNGS) is rapidly transitioning from research to clinical practice. It is a comprehensive analysis of microbial and host genetic material (DNA and RNA) in patients’ samples. The mNGS can detect all potential pathogens, and has been used for diagnosis of toxoplasmosis [7, 9].

Most patients with cerebral toxoplasmosis following allo-HSCT are believed to result from reactivation of latent infection, with reported mortality rates as high as 80% [10]. Reactivation of cerebral toxoplasmosis following allo-HSCT has been associated with severe immunosuppression, delayed recovery of toxoplasma-specific cellular response, and severe graft versus host disease (GVHD) [8, 11]. Risk factors for increased mortality from post-transplant toxoplasmosis include delayed diagnosis, disseminated toxoplasmosis as opposed to isolated brain involvement, anti-thymocyte globulin-induced T cell depletion, and poor compliance with prevention and treatment [10, 11].

Allo-HSCT has been an important approach for treating transfusion-dependent β-thalassemia (β-TM). Transplantation from a human leukocyte antigen (HLA) matched sibling donor during childhood (under 12 years old) has been considered the gold standard treatment for severe β-TM [12, 13]. Allo-HSCT is not the only curative treatment option for transfusion-dependent β-TM. A new Food and Drug Administration (FDA)ーapproved one-time infusion of beti-cel is the first potentially curative gene therapy for transfusion-dependent β-TM [14]. However, patients after allo-HSCT may have false negative toxoplasma antibody tests due to the immunosuppressive effects of allo-HSCT [6, 15], which may mislead clinical diagnosis and treatment. Additionally, the clinical symptoms and neuroimaging findings of cerebral toxoplasmosis and primary central nervous system lymphoma (PCNSL) partially overlap, making timely and accurate diagnosis challenging and contributing to a delayed diagnosis of cerebral toxoplasmosis [16].

There is limited literature available regarding cerebral toxoplasmosis in children with severe β-TM after allo-HSCT. Furthermore, reports on the neuroimaging findings of cerebral toxoplasmosis are scarce. Due to the severity of this complication and the limited information available in pediatric transplant population, it is prudent to enhance our understanding of its neuroimaging and clinical features to assist in early diagnosis and treatment. Here, we retrospectively analyze three patients with cerebral toxoplasmosis after allo-HSCT for severe β-TM. We also reviewed the literature and summarized the neuroimaging findings of cerebral toxoplasmosis.

## Materials and methods

We searched medical records in our hospital for all patients with severe β-TM who underwent allo-HSCT from September 2020 to April 2022 and identified three patients with severe β-TM who had cerebral toxoplasmosis after allo-HSCT. The clinical data of the patients, including symptoms, underlying conditions, neuroimaging features, mNGS results, and treatment strategies, were abstracted from the medical records. The neuroimaging features assessed for each patient included the location, number, and size of the brain lesions, as well as the computed tomography (CT) density or magnetic resonance imaging (MRI) signal characteristics, enhancement patterns, and surrounding edema.

Additionally, we conducted a comprehensive search for relevant literature using the following terms in databases including PubMed, Embase, Web of Science, Elsevier (ScienceDirect), Ovid, Emerald, and China National Knowledge Infrastructure (CNKI): ‘Toxoplasma’ or ‘Toxoplasmosis’ or ‘Cerebral toxoplasmosis’ only, or combined with ‘Hematopoietic stem cell transplantation (HSCT)’ or ‘Hematopoietic stem cells’ or ‘allo-HSCT’ or ‘β-Thalassemia’ or ‘Thalassemia’ or ‘mNGS’ or ‘Magnetic Resonance Imaging’ or ‘Computed Tomography’.

### Ethical statement

The Institutional Review Board (IRB) of the First Affiliated Hospital of Guangxi Medical University approved the study (IRB: 2024-E234-01).

## Results

### Case series

#### Patient information

Relevant diagnostic and treatment information for the three patients of cerebral toxoplasmosis following allo-HSCT in children with severe β-TM are presented in Table 1. The three patients were all male, between 6 to 10 years of age with a median age of 9 years, and all of them had transfusion-dependent β-TM. Patient 1 had fever, dizziness, vomiting, and fatigue on day 88 after allo-HSCT; Patient 2 had fever, chills, joint pain, right deviation of the tongue, flattening of the right nasolabial fold, and coma on day 36 after allo-HSCT; Patient 3 had fever, headache, nausea, involuntary tremor of the left lower limb, and involuntary flexion of the fingers of the left hand on day 99 after allo-HSCT. All three children had the common symptom of fever and GVHD after allo-HSCT.

**Table 1:** Patient characteristics, clinical presentation and outcome for this case series

**Patient no.**	**1**	**2**	**3**
Age (years)/sex	9/Male	6/Male	10/Male
Underlying disease	Severe β-thalassemia	Severe β-thalassemia	Severe β-thalassemia
Transfusion history	Yes	Yes	Yes
Donor type	HLA 10/10-identical sibling	HLA 6/12-identical sibling	HLA-identical sibling
Pre-transplant serum toxoplasma antibodies	ND	ND	ND
Prevention of toxoplasmosis using TMP/SMT	No	No	No
Pre-transplant conditioning regimen	BU+CY+FLU+ATG	BU+CY+FLU+ATG	BU+CY+FLU+ATG
Protocol for preventing GVHD after transplantation	MTX+MMF+CsA	FK506+MTX+MMF+Basiliximab	CsA+MMF+MTX
GVHD	Yes	Yes	Yes
Main symptoms	Fever, dizziness, vomiting, fatigue	Fever, chills, joint pain, tongue extension right crooked, right nasolabial sulcus shallow, coma	Headache, fever, nausea, involuntary shaking of the left lower limb and involuntary flexion of the left finger
Time after transplant when symptoms began to appear	88 days	36 days	99 days
Blood and CSF toxoplasma antibodies	Serum IgG: +, serum IgM, CSF IgG, IgM: −	Serum and CSF IgG, IgM: −	CSF IgM: +, CSF IgG, serum IgG, IgM: −
Blood and CSF mNGS	Blood: 1077, CSF: 7263	Blood: 151, CSF: 34183	Blood: 262, CSF: 58412
Treatment	Azithromycin, co-trimoxazole, clindamycin	No	Azithromycin, cotrimoxazole
Outcome	Improved without recurrence	Death	After 5 months of treatment, symptoms recurred one month after self-withdrawal, and were improved by resuming treatment
Time from onset of symptoms to the end	120 days	26 days	300 days
Neuroimaging follow-up	Brain MRI: brain lesion decreased in size.	ND	Brain MRI: brain lesions mostly resolved.

ATG: Anti-human thymocyte globulin; BU: Busulfan; CsA: Cyclosporin A; CSF: Cerebrospinal fluid; CY: Cyclophosphamide; FK506: Tacrolimus; FLU: Fludarab; GVHD: Graft-versus-host disease; HLA: Human leukocyte antigen; MMF: Mycophenolate mofetil; mNGS: Metagenomic next generation sequencing; MRI: Magnetic resonance imaging; MTX: Methotrexate; ND: Not done; TMP/SMT: Trimethoprim/sulfamethoxazole.

#### Laboratory test results

Patient 1 had positivity for anti-toxoplasma IgG antibodies in serum but negative for CSF antibodies, while Patient 3 had positivity for anti-toxoplasma IgM antibodies in the CSF but negative for serum antibodies in conventional laboratory testing. Patient 2 tested negative for both serum and CSF antibodies. However, mNGS showed positivity for toxoplasma in both blood and CSF for all three patients (Table 1).

#### Neuroimaging findings

All three patients underwent brain MRI scans. In addition, Patient 3 underwent brain magnetic resonance spectroscopy (MRS), and a 2-deoxy-2-[fluorine-18] fluorodeoxyglucose positron emission tomography/computed tomography (^18^F-FDG PET/CT) scan (Table 2). Patient 1 had a brain CT scan while Patients 2 and 3 did not undergo brain CT scan.

**Table 2:** Neuroimaging findings in patients with cerebral toxoplasmosis after allogeneic hematopoietic stem cell transplantation (allo-HSCT) for this case series

**Patient no.**	**Location of brain lesions**	**Number of lesions**	**Neuroimaging examination**	**Neuroimaging findings**
1	Left frontal lobe, right temporal lobe	2	Non-contrast head CT, brain MRI, MRS	*Head CT:* A nodular slightly low-density lesion in the left frontal lobe measuring 11 mm in size. An irregular small patchy low-density lesion in right temporal lobe. *Brain MRI:* Left frontal lobe lesion showing a three-layered target sign of low-high-low signal intensity lesion, a ring-shaped hypointense signal on DWI and mild enhancement. The right temporal lobe showing a small patchy lesion with a blurry margin, with no restricted diffusion and mild enhancement in a ring shape.
2	Bilateral greater cerebellar hemispheres, basal ganglia, thalamus	Multiple	Non-contrast brain MRI	*Brain MRI:* Innumerable small (less than 5 mm in size) nodular T1 hypointense & T2 hyperintense lesions, some showing mild to moderate restricted diffusion, some with peripheral ring-shaped high signal and central low signal.
3	Right peduncle, right temporal lobe	2	Brain MRI, MRS, PET/CT	*Brain MRI:* Lesions in the right cerebral peduncle and right temporal lobe measuring up to 23 mm in size showing iso-low signals on T1 and T2, with a three or four layered target sign of low-high-low-high signals from the periphery to the center, with peripheral ring-shaped high signals and central low signals on DWI, and with a distinct ring-shaped and eccentric core, as well as central core target sign enhancement. *MRS:* Significant decrease in NAA (N-acetylaspartate) peak, varying degrees of reduction in Creatine (Cr) and Choline (Cho) peaks, increased Cho/Cr ratio, and bidirectional elevation of lactate (Lac) and lipid (Lip) peaks. *Brain PET/CT:* Reduced PET activity in the right cerebral peduncle lesion, and decreased central uptake with slight peripheral increase of PET activity in the right temporal lobe lesion.

CT: Computed tomography; DWI: Diffusion weighted imaging; FLAIR: Fluid attenuated inversion recovery; mm: Millimeter; MRI: Magnetic resonance imaging; MRS: Magnetic resonance spectroscopy; PET/CT: Positron emission tomography/computed tomography; T1: T1-weighted imaging; T2: T2-weighted imaging.

**Figure 1.**
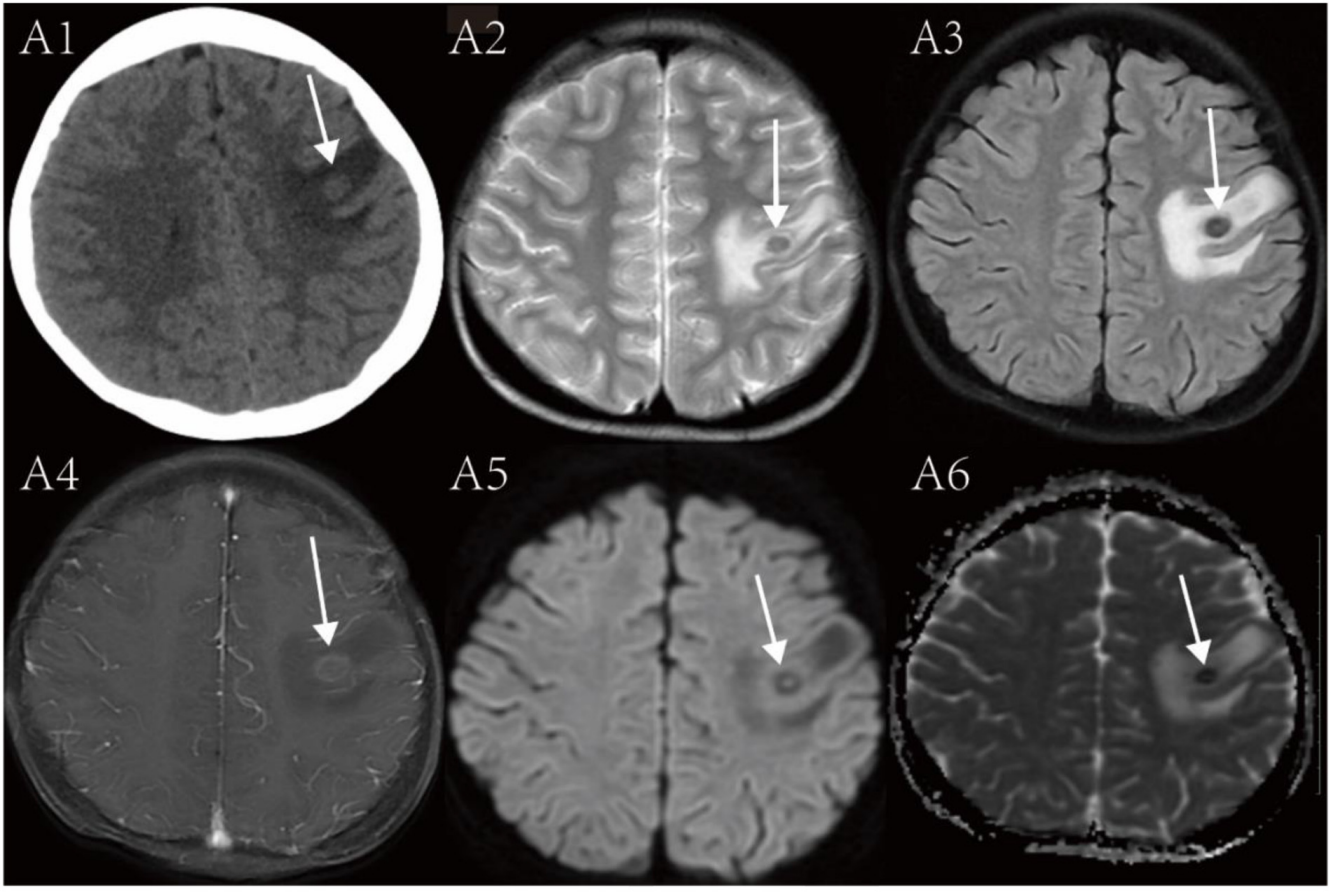
**Brain imaging findings for patient 1 upon presentation.** (A1) Non-contrast CT image showing a slightly low-density nodular lesion in the left frontal lobe on (white arrow) with surrounding low-density edema. (A2) T2-weighted image showing the left frontal lobe lesion with a three-layered target sign of low-high-low signal from the periphery to the center as seen on (white arrow). (A3) T2-fluid attenuated inversion recovery (FLAIR) image showing a nodular central punctate high signal surrounded by low signal in the left frontal lobe as seen on (white arrow). (A4) The contrast-enhanced T1 image showing the ring enhancement of the lesion (white arrow). (A5) Diffusion weighted imaging (DWI) showing the left frontal lobe lesion with a central dot of isointensity surrounded by a ring of hypointensity and then a ring of isointensity. (A6) Apparent diffusion coefficient (ADC) image showing the left frontal lobe lesion with a central point of high signal surrounded by a ring of low signal with associated edema.

The brain CT scan for Patient 1 showed a nodular, slightly low-density 1.1 cm size lesion in the left frontal lobe with clear margins (Figure 1: A1). The brain MRI for Patient 1 showed the left frontal lobe lesion having a mixed signal on T1-weighted imaging (T1WI), a target appearance on T2-weighted imaging (T2WI) and T2 fluid-attenuated inversion recovery (T2-FLAIR) and mild enhancement in a ring-like pattern (Figure 1: A2-A4). Patient 1 also had another lesion which was located in the right temporal lobe, and its MRI findings are presented in Figure S1. Follow-up brain MRI for Patient 1 after four months of treatment showed the left frontal lobe lesion being decreased in size and decreased associated edema (Figure S2).

**Figure 2.**
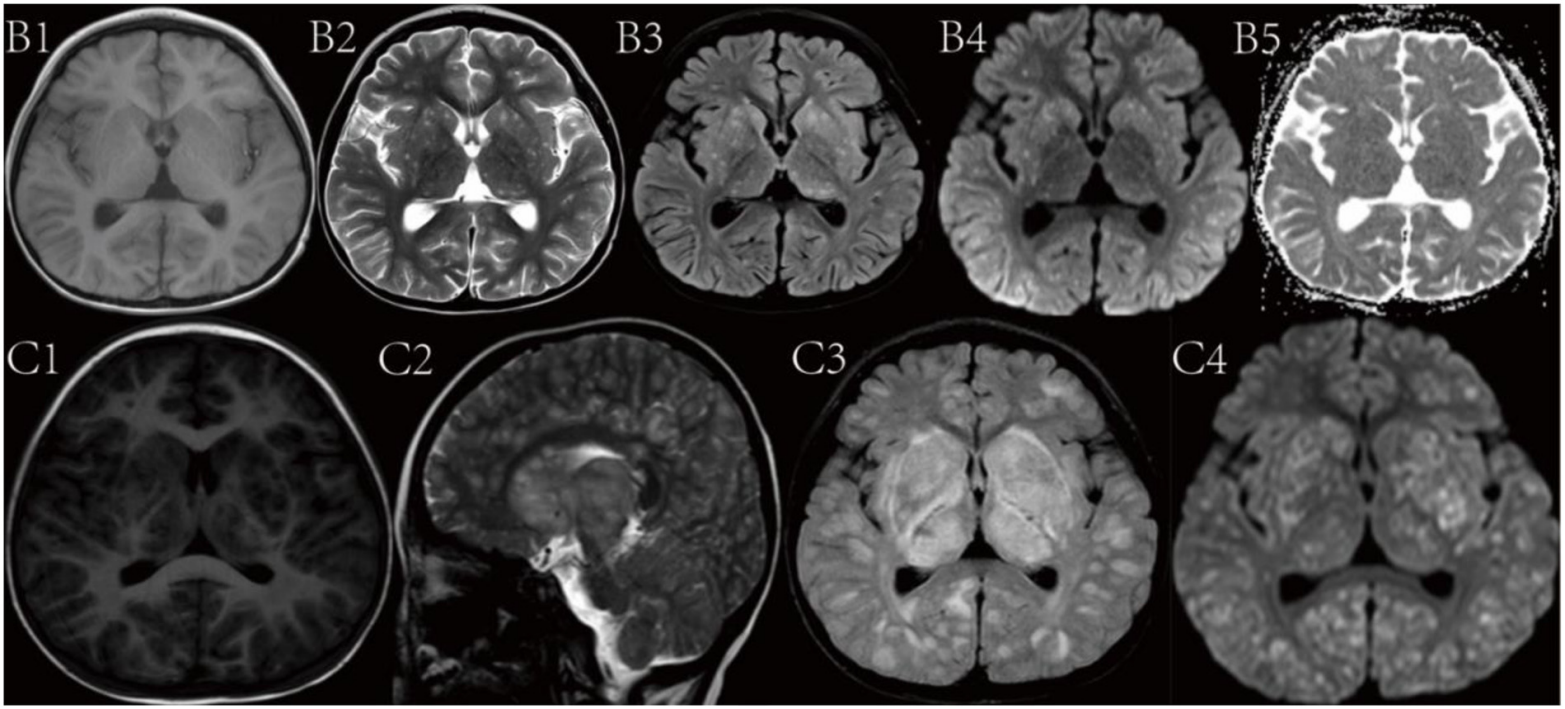
**Brain magnetic resonance imaging findings for patient 2 upon initial presentation at +54 days of transplant (B1-B5) and 6 days later at +60 days of transplant (C1-C4).** Images from B1 to B5 showing multiple punctate and small nodular lesions less than 0.5 cm in size in bilateral cerebral hemispheres, basal ganglia, and thalamus, with indistinct margins upon presentation. Images from C1 to C4 showing worsening lesions with significant increase in size and number of the brain lesions. Brain magnetic resonance imaging including the T1-weighted (B1, C1), T2-weighted (B2, C2), T2-fluid attenuation inversion recovery (FLAIR, B3, C3), diffusion-weighted imaging (DWI, B4, C4), and apparent diffusion coefficient images (ADC, B5).

Brain MRI scan for Patient 2 showed multiple scattered punctate and nodular lesions in bilateral cerebral hemispheres including basal ganglia, and thalami upon presentation. The lesions appeared as iso- or slightly low signal on T1WI, high signal with blurred margins on T2WI, and iso- or slightly high signal on T2-FLAIR. Some lesions showed mild-to-moderate restricted diffusion on DWI, while others exhibited peripheral ring-like high signal and central low signal on DWI. The lesions were slightly high signal on the ADC (Figure 2: B1-B5). A follow-up brain MRI obtained 6 days after presentation showed a significant increase in both the number and size of the lesions throughout the supratentorial brain (Figure 2: C1-C4).

**Figure 3.**
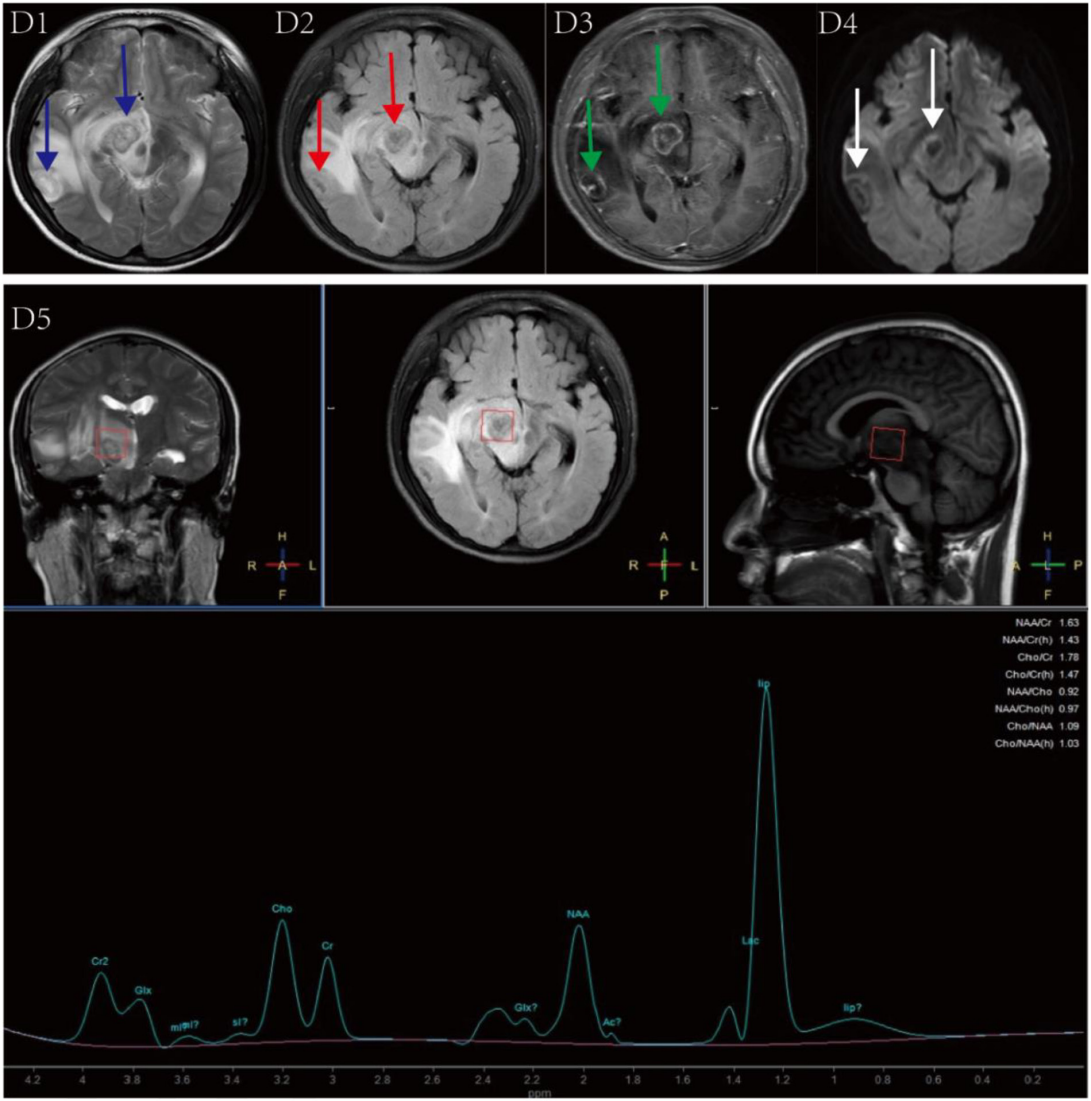
**Brain magnetic resonance imaging (MRI) findings for patient 3 upon presentation at +107 days of transplant**. (D1, D2) The T2-weighted and T2-fluid attenuation inversion recovery (FLAIR) images showing lesions in the right temporal lobe and right cerebral peduncle with a three or four-layered target sign of low-high-low-high signal from the periphery to the center (blue and red arrows). (D3) Post-contrast MRI image showing the eccentric core enhancement, as well as central core target-like enhancement within the lesions (green arrow). (D4) Diffusion-weighted imaging (DWI) showing low signal intensity in the central area and isointensity or slightly hyperintensity in the peripheral ring (white arrow). (D5) Magnetic resonance spectroscopy showing decreased Choline (Cho), Creatine (Cr) and N-acetyl aspartate (NAA) peaks, elevated Cho/Cr ratio, decreased NAA/Cho ratio, and increased lactic acid (Lac) and lipid (Lip) peaks in the right cerebral peduncle lesion.

**Figure 4.**
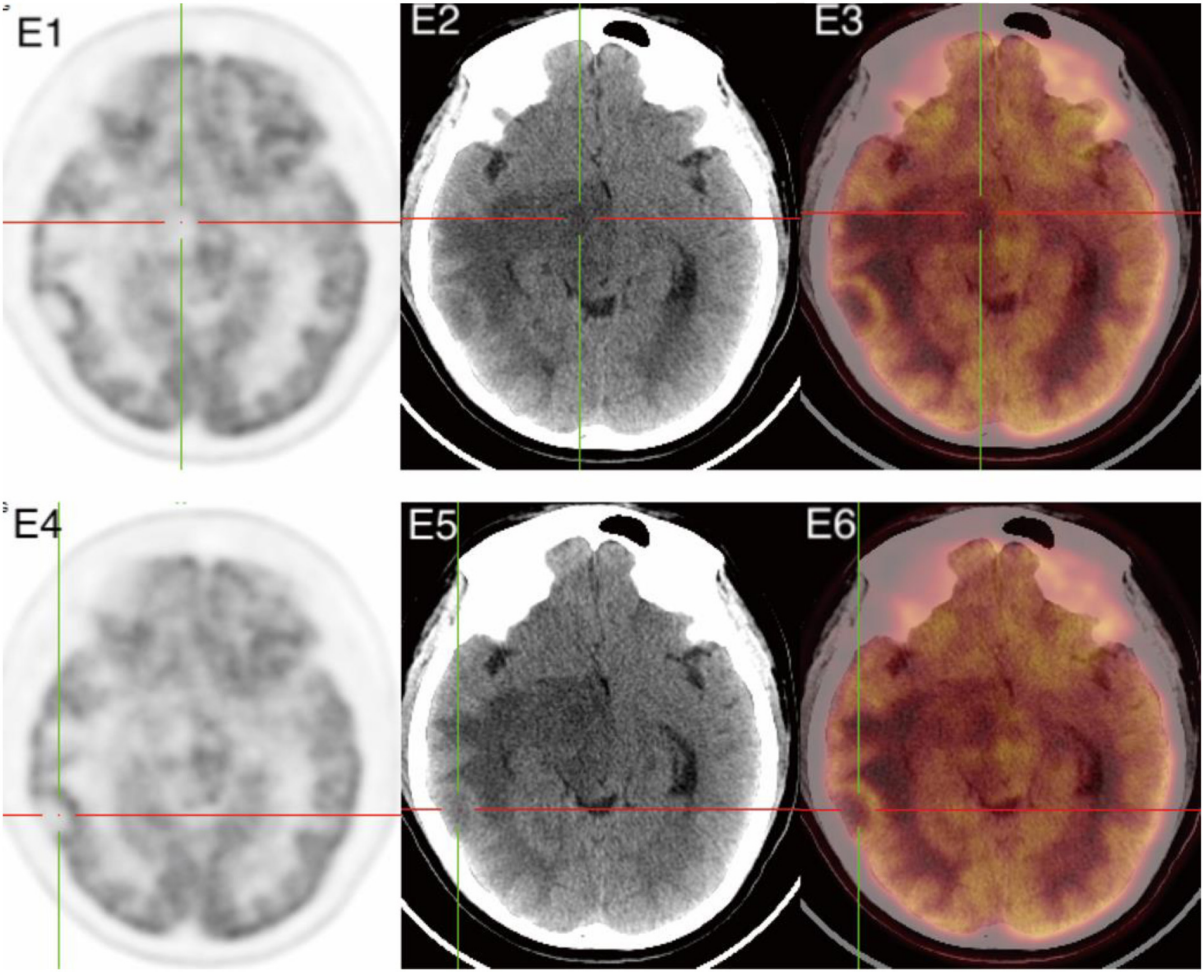
**18F-FDG PET/CT images for patient 3**. (E1-E3) Reduced radiotracer uptake in the right cerebral peduncle lesion on the FDG PET (E1) image, CT image (E2), and PET/CT fusion image (E3) at the intersection of the red and green lines. (E4-E6) Decreased central radiotracer uptake and slightly increased peripheral uptake in the right temporal lobe lesion on the FDG PET image (E4), CT image (E5), and PET/CT fusion image (E6) at the intersection of the red and green lines.

Brain MRI for Patient 3 showed two lesions with heterogeneous signal intensity in the right temporal lobe and the right cerebral peduncle. These lesions appeared iso- or low signal on T1WI, a target appearance with a three-layered or four-layered low-high-low-high signal pattern on T2WI and T2-FLAIR, and a ring- and target-like enhancement (Figure 3: D1-D3), and its DWI findings are presented in the (Figure 3: D4). MRS showed a significant decrease in the N-acetylaspartate (NAA) peak in the right cerebral peduncle lesion, with varying degrees of reduction in the Creatine (Cr) and Choline (Cho) peaks, an increased Cho/Cr ratio, and bidirectional changes in lactic acid (Lac) and lipid (Lip) peaks (Figure 3: D5). Furthermore, the PET/CT scan for Patient 3 showed decreased radiotracer uptake within both lesions (Figure 4). However, there was a slight increase in radiotracer uptake around the lesion in the right temporal lobe (Figure 4). Both lesions were surrounded by varying degrees of edema. Follow-up brain MRI for Patient 3 after five months of treatment showed the lesions in the right cerebral peduncle and right temporal lobe being decreased in size and associated edema (Figure S3).

#### Treatment and prognosis

Patient 1 had improvement of symptoms with no recurrence after four months of treatment with azithromycin, sulfamethoxazole, and clarithromycin palmitate dispersible tablets, and his follow-up brain MRI is presented in Figure S2 showing the brain lesion decreased in size. Patient 2 had a delayed diagnosis due to non-specific neuroimaging features, negative serum toxoplasma antibody test, and a positive mNGS report that was not completed until post-mortem. As a result, the patient did not receive anti-toxoplasma treatment, and his condition rapidly progressed to acute disseminated toxoplasma encephalitis, ultimately resulting in his death. Patient 3 had clinical improvement after intermittent use of azithromycin and sulfamethoxazole for five months, and his follow-up brain MRI is presented in Figure S3 showing lesions decreased in size. However, he later experienced a relapse of cerebral toxoplasmosis after discontinuing the medication for one month. After one month of treatment, his symptoms improved (Table 1).

### Search results

A total of 18 articles were identified through literature search regarding toxoplasmosis infection in patients who had been treated for underlying diseases other than β-TM. Together, these articles described clinical information and treatment strategies for 23 patients, consisting of 14 males, 8 females and 1 patient with unspecified gender, with most of the patients undergoing transplantation (Table 3). The median age of the patients from the literature was 33 years, with a range of 6–68 years. The patients identified from literature had the following underlying diseases: myelodysplastic syndrome (2 patients) [17], leukemia (12 patients) [7, 15, 17–24], non-Hodgkin’s lymphoma (1 patient) [17], diffuse large B-cell lymphoma (1 patient) [25], Fanconi anemia (1 patient) [26], severe aplastic anemia (1 patient) [6], multiple myeloma (2 patients) [20], and AIDS (3 patients) [27–29]. Nineteen out of these 23 patients from literature underwent allo-HSCT, and one patient underwent cord blood transplantation. The remaining three patients with AIDS did not undergo transplantation. Prior to transplantation, 11 out of the 23 patients from the literature (55%) had positivity for serum anti-toxoplasma antibodies, and 8 patients received medication to prevent toxoplasma infection. The time from HSCT to symptom onset ranged from 22 to 390 days, with a median time of 90 days.

Among the 23 patients identified in the literature who had cerebral toxoplasmosis after HSCT for other underlying diseases, the main symptoms were fever (60.9%), headache (47.8%), altered consciousness (21.7%), and neurological manifestations such as seizures, limb weakness, ataxia, sluggish pupillary response, and hemianopia.

Diagnostic methods for these patients from literature included brain MRI, brain CT, PCR examination of CSF and/or blood, toxoplasma gene detection in blood or CSF, mNGS of blood sample, pathological examinations and testing of serum and/or CSF anti-toxoplasma antibodies. Additional tests included May-Grünwald Giemsa staining of CSF, bone marrow smear examination, flow cytometry, fundus examination, and pathogen culture of CSF. Toxoplasma infection was found in various sites, with the brain being the most common (20 patients, 87.0%, including 15 patients with isolated brain infection); followed by the heart (3 patients, 13.0%); eyes, lungs, and adrenal glands (2 patients each, 8.7%); and liver, pancreas, stomach, bone marrow, and subcutaneous tissue (1 patient each, 4.3%). Concurrent infection in two sites was observed in two patients (8.7%), and infection in multiple sites was observed in three patients (13.0%). Anti-toxoplasma treatment was administered to 20 out of the 23 patients identified in literature. Following treatment, symptom improvement was observed in 14 patients (60.7%), while 9 patients (39.1%) died. Among the deceased, 3 patients were attributed to disseminated toxoplasmosis, 5 patients to isolated cerebral toxoplasmosis, and 1 patient to both the brain and heart being infected with toxoplasmosis.

To broaden our understanding of cerebral toxoplasmosis, we also reviewed literature encompassing neuroimaging features in patients having cerebral toxoplasmosis with and without transplantation. We identified a total of 78 patients from 10 publications [18, 21, 22, 27, 30–35] including four papers already included in Table 3 and additional six different papers. This comprehensive neuroimaging review on cerebral toxoplasmosis is presented in Table S1. Out of the 78 patients, one patient had two lesions, 56 patients had multiple lesions, and the number of lesions was not specified in 21 patients. The lesions were located in the cerebrum (57 patients, 73.1%), cerebellum (50 patients, 64.1%), basal ganglia (45 patients, 57.7%), thalamus (4 patients, 5.1%), midbrain (2 patients, 2.6%), internal capsule, hippocampus, and supratentorial and infratentorial brain parenchyma (1 patient in each, 1.3%). Brain CT scans revealed low density lesions in 4 patients, ring enhancement in 24 patients, and no enhancement in 3 patients. On T1WI, 11 patients showed iso- or hypointensity within the brain lesions, and one patient showed lesions with peripheral hyperintensity and central hypointensity. On T2WI, one patient showed high signal intensity lesions, 16 patients showed lesions with a target sign, displaying concentric layers of low-high-low signal intensity from the outer to the inner region, 10 patients showed deep lesions with high signal intensity or heterogeneous signal, and 50% of superficial lesions showed low signal intensity. On T2-FLAIR, 17 patients exhibited lesions with a target sign, displaying concentric layers of low-high-low signal intensity from the outer to the inner region, 11 patients showed lesions with heterogeneous, iso-, hypo-, or hyperintense signals, and 2 patients showed lesions with high signal intensity. Perilesional edema was observed in 11 patients. On DWI, 2 patients with multiple lesions showed outer high and inner low signal. The ^18^F-FDG PET/CT showed decreased uptake in six patients. MRS showed increased Cho/Cr in 11 patients and normal Cho/Cr in four patients.

**Table 3:** Summary of the 23 patients from the literature who had toxoplasmosis after treatment for underlying conditions other than β-thalassemia

	**Patients**										
**No.**	**1**	**2**	**3**	**4**	**5**	**6**	**7**	**8**	**9**	**10**	**11**
Age (years)/sex	17/unknown	13/M	17/F	12/F	65/F	56/M	68/F	34/M	22/M	63/M	52/M
Underlying disease	FA	SAA	Ph+ ALL	AML	MM	MM	AML	CML	AML	AMMOL	AML
Donor type	HLA-matched unrelated donor	HLA-matched unrelated donor	HLA-matched unrelated donor	Haplo-identical mother	HLA-identical sibling	HLA-matched unrelated donor	Mismatched unrelated donor	unknown	HLA-matched unrelated donor	HLA-matched unrelated donor	HLA-matched unrelated donor
Pre-transplant serum toxoplasma antibodies	ND	ND	ND	IgG: +	IgG: +	IgG: +	+	ND	IgG: +	IgG: +	Unknown
Prevention of toxoplasmosis using TMP/SMT	No	No	No	Atovaquone	Unknown	Unknown	Yes	No	Unknown	Yes	Yes
Cardinal symptom	Fever, headache, visual impairment	Fever, headache, disturbance of consciousness (drowsiness), nausea	Fever, headache, nausea, seizures	Headache, vomiting, seizures	Symptoms of multiple organ failure	Fever	Fever, neurological and psychological changes, seizures	Fever, headache, right hemianopsia	Fever, confusion and memory loss	Seizures and right limb weakness	Disturbance of consciousness (drowsiness), disorientation, loss of short-term memory
Time after transplant when symptoms began to appear	90 d	270 d	58 d	150 d	Unknown	90 d	390 d	Unknown	60 d	120 d	78 d
Diagnostic procedures	Serum and CSF toxoplasma IgG and IgM, brain CT and MRI: +	Plasma toxoplasma DNA and CSF PCR, brain CT and MRI: +	Toxoplasma DNA in CSF and brain MRI: +	Serum toxoplasma IgG, blood and CSF PCR, brain CT and MRI:+	Postmortem histological examination	Postmortem histological examination	Serum toxoplasma IgM, CSF PCR, brain MRI: +	CSF PCR, brain MRI:+	Serum toxoplasma IgG and IgM, brain MRI, postmortem histological examination: +	Serum toxoplasma IgG, brain MRI, brain biopsy: +	CSF etiological culture and PCR, brain MRI: +
Infection site	Brain, eye	Brain	Brain	Brain	Brain, lungs, stomach, heart muscle, adrenal glands and subcutaneous tissue	Brain, liver, lungs, adrenal glands, heart, bone marrow and pancreas	Brain	Brain	Brain	Brain	Brain
Treatment	Clindamycin, trimethoprim, steroid hormones	Pyrimethamine, sulfadiazine, clindamycin, dexamethasone	Compound tramozole, clindamycin	Pyrimethamine, sulfadiazine and leucovorin	No	No	Pyrimethamine, clindamycin, sulfadiazine	TMP/SMT	Sulfadiazine and pyrimethamine	Sulfadiazine and pyrimethamine	Compound triazole, Clindamycin, co-trimoxazole, pyrimethamine, sulfadiazine and leucovorin
Outcome	Improved	Improved	Improved	Improved	Death	Death	Death	Improved	Death	Improved	Improved
Time from onset of symptoms to the end	180 d	150 d	180 d	60 d	63 d	139 d	75 d	Unknown	33 d	Unknown	150 d
References	[26]	[6]	[18]	[19]	[20]	[20]	[20]	[21]	[15]	[15]	[22]
Age (years)/sex	54/F	65/M	35/M	33/M	10/M	33/F	9/M	16/M	14/F	6/M	12/M	34/F
Underlying disease	CML	CMML	AIDS	AIDS and HPS	AML M7	AIDS and HPS	DLBCL	MDS	AML	NHL	MDS	ALL
Donor type	HLA-identical sibling	UCBT	No	No	HLA-matched father	No	HLA-identical sibling	HLA-matched unrelated donor	Partial HLA-matched family donors	HLA-matched unrelated donor	HLA-matched family donor	Haplo-identical daughter
Pre-transplant serum toxoplasma antibodies	Unknown	+	ND	ND	ND	ND	ND	IgG: +	IgG: +	IgG: +	IgG: +	ND
Prevention of toxoplasmosis using TMP/SMT	Yes	Fluconazole	No	No	No	No	No	Yes	Yes	No	No	No
Cardinal symptom	Headache and neck stiffness	Headache and fever	Headache, right side pupil unresponsiveness	Fever, difficulty breathing	Fever	Fever, disturbance of consciousness	Fever, lower limb weakness, ataxia, disturbance of consciousness (drowsiness)	Headache	Fever, headache, vomiting	Disturbance of consciousness (drowsiness), respiratory distress syndrome, vomiting and seizures	Impairment of vision	Fever, headache, dizziness, lethargy, mental decline, visual field loss
Time after transplant when symptoms began to appear	234 d	37 d	No	No	22 d	No	Unknown	266 d	55 d	55 d	Pretransplant	42 d
Diagnostic procedures	CSF PCR, brain MRI: +	May-Grünwald Giemsa staining of CSF and CSF PCR: +	Serum toxoplasma antibodies, brain CT and MRI, histopathological examination: +	Serum toxoplasma antibodies, blood mNGS: +	Serum toxoplasma IgG, blood mNGS, bone marrow smear test, blood flow cytometry: +	Serum toxoplasma IgG, blood and CSF PCR, brain MRI: +	Brain MRI, brain biopsy: +	Serum toxoplasma IgG, blood PCR, brain CT and MRI: +	Serum toxoplasma IgG, blood PCR, brain CT and MRI: +	Serum toxoplasma IgG, blood PCR, brain CT and MRI, brain biopsy: +	Serum toxoplasma IgG, IGM, fundus examination: +	CSF toxoplasma IgG, CSF mNGS, brain MRI: +
Infection site	Brain	Brain	Brain and heart	Unknown	Unknown	Brain	Brain	Brain	Brain	Diffuse toxoplasmosis	Retina	Brain
Treatment	Clindamycin, co-trimoxazole	Pyrimethamine, sulfadiazine	No	Sulfamethoxazole, trimethoprim, methylprednisolone, clindamycin	TMP, SMT, clindamycin	TMP, SMT	TMP, SMT	Cotrimoxazole, azithromycin (4 w)	Sulfamethoxazole (7 d)	Cotrimoxazole + azithromycin (3 d), pyrimethamine + sulfadiazine (5 w)	Pyrimethamine + sulfadiazine (4 w)	TMP, SMT, clindamycin
Outcome	Death	Death	Death	Improved	Improved	Improved	Improved	Death	Improved	Death	Improved	Improved
Time from onset of symptoms to the end	248 d	82 d	Unknown	120 d	270 d	1095 d	730 d	312 d	275 d	58 d	1542 d	120 d
References	[22]	[23]	[27]	[28]	[7]	[29]	[25]	[17]	[17]	[17]	[17]	[24]

AIDS: Acquired immune deficiency syndrome; AML: Acute myelogenous leukemia; AMMOL: Myelomonocytic leukemia; CML: Chronic myeloid leukemia; Cmml: Chronic myelomonocytic leukemia; CSF: Cerebrospinal fluid; CT: Computed tomography; d: Days; DLBCL: Diffuse large B-cell lymphoma; DNA: Deoxyribonucleic acid; F: Female; FA: Fanconi anemia; HLA: Human leukocyte antigen; HPS: Hemophagocytic syndrome; M: Male; MDS: Myelodysplastic syndromes; MM: Multiple myeloma; MRI: Magnetic resonance imaging; ND: Not done; NHL: Non-Hodgkin lymphoma; PCR: Polymerase chain reaction; Ph+ ALL: Philadelphia chromosome-positive acute lymphoblastic leukemia; SAA: Severe aplastic anemia; TMP/SMT: Trimethoprim/sulfamethoxazole; UCBT: Umbilical cord blood transplantation; w: Weeks.

## Discussion

In this case series, the three children with severe β-TM who had cerebral toxoplasmosis after allo-HSCT presented with fever (3 of 3 children), headache (1 of 3 children), altered consciousness (1 of 3 children), and neurological deficits (2 of 3 children). Among the 23 patients identified in the literature who had cerebral toxoplasmosis for underlying diseases other than β-TM also had fever as a common symptom. Overall, we found that the clinical manifestations of cerebral toxoplasmosis after allo-HSCT in children with severe β-TM were similar to those after HSCT for underlying diseases other than β-TM.

However, we found that some patients tested negative for toxoplasma antibodies. This may be due to immunosuppression, which prevented patients from producing sufficient antibodies, indicating inadequate immune reconstitution of the patients, as previously reported [6, 15].

Neuroimaging is essential for diagnosing and predicting the outcome of cerebral toxoplasmosis. Our literature review focusing on neuroimaging findings of cerebral toxoplasmosis found that cerebral toxoplasma lesions were mainly located in the cerebrum (73.1%), cerebellum (64.1%), and basal ganglia (57.7%), with a smaller proportion affecting the thalamus (5.1%) and brainstem (2.6%) (Table S1). It is prudent to note that all known patients from the literature presented with multifocal brain lesions (≥2 lesions) of varying sizes. Previous studies have reported that cerebral toxoplasmosis in patients who have undergone HSCT typically manifested as multifocal brain lesions, with the basal ganglia being the most commonly affected region [8, 36]. The present case series on cerebral toxoplasmosis after allo-HSCT for pediatric patients with severe β-TM showed two of the three patients (Patient 2 and Patient 3) having lesions involving subcortical brain lesions such as basal ganglia and cerebral peduncle extending to thalamus. On the other hand, cerebral toxoplasmosis lesions after allo HSCT for patients with other underlying diseases tend to have lesions more often in the brain lobar regions than in the basal ganglia [18]. There seemed to be a differential distribution of cerebral toxoplasmosis lesions after allo-HSCT between pediatric patients with severe β-TM and those with other underlying diseases. However, our case series was too small to ascertain a definitive differential distribution. Further research with a larger sample size is needed to assess the distribution pattern of brain lesions in patients undergoing allo HSCT for various underlying diseases.

Typical CT manifestations of cerebral toxoplasmosis include lesions with low density, ring enhancement post-contrast administration, and/or a ‘target sign’ [27, 30]. Also, T1-weighted MRI often shows ring enhancement and/or eccentric or target sign enhancement as reported [2, 30]. The CT scans of the patients in this study revealed lesions with slightly low or low density, consistent with the previously reported CT imaging findings of cerebral toxoplasmosis [27]. In our case series and review of the literature, we found that the majority of patients showed circular enhancement on CT and MRI T1-enhanced scans, and some patients showed typical eccentric or central target-like enhancement on T1-enhanced scans. A previous report [36] has suggested that the eccentric or central target-like enhancement is a specific imaging feature of cerebral toxoplasmosis in patients with AIDS and other immunocompromised conditions. Here, we identified similar neuroimaging findings of brain lesions with target-like enhancement in children with cerebral toxoplasmosis after allo-HSCT for severe β-TM.

In T2WI or T2-FLAIR imaging, a target sign was observed in the brains of two children (Patient 1 and Patient 3) with severe β-TM and cerebral toxoplasmosis in our case series. Of the patients we reviewed in literature, we identified 18 patients with similar findings (Table S1). Literature has reported the target sign as an auxiliary neuroimaging feature for cerebral toxoplasmosis, which helps distinguish it from PCNSL [7]. PCNSL typically presents as a mass lesion with either equal or high density on non-enhanced CT scans, owing to its rich cellular characteristics [37], while lesions with cerebral toxoplasmosis showed hypodensity on CT.

Patient 2 showed worsening of multiple high T2WI signal lesions within the brain, indicating diffuse toxoplasma encephalitis, likely due to hematogenous dissemination, as previously reported by Lee et al. [27]. It has been reported that disseminated toxoplasmosis is associated with high mortality resulting in death of three patients as indicated in the literature review in Table 3.

In immunosuppressed patients, Toxoplasma gondii initially causes necrotic lesions in the brain with a minimal inflammatory response, followed by infiltration of lipid-laden macrophages, ultimately leading to abscess formation [31]. In Patient 3, MRS showed reduced Cho and NAA peaks in the brain lesions, indicating decreased cellular synthesis and diminished neuronal integrity. In addition, the increased lactate peaks indicated increased anaerobic metabolism from tissue necrosis in toxoplasmosis lesions. PCNSL, on the other hand, is relatively cellular, forming an infiltrative lesion mixing the central core of tumor cells with the original brain parenchyma [38], leading to an expected increase in the Cho leak. This difference could help to differentiate between the cerebral toxoplasmosis and lymphoma. An increase in the Cho/Cr ratio was observed in Patient 3, and a literature review found similar MRS findings in 11 patients of cerebral toxoplasmosis. Chinn et al. [32] suggested that relatively immature toxoplasmosis lesions may have considerable cellularity with little or no necrosis or macrophage infiltration, resulting in a spectrum with a relative increase in Cho, thus the increased Cho/Cr ratio as noted from our Patient 3.

Patient 3 in our series had reduced radiotracer uptake in the FDG PET/CT scan in two lesions, with one lesion showing slightly increased peripheral radioactive uptake. Our literature review focusing on neuroimaging features also showed reduced FDG uptake in six patients of cerebral toxoplasmosis (Table S1). A prior study showed that infectious processes such as cerebral toxoplasmosis was associated with reduced metabolic activity as compared to the contralateral normal brain [37]. This may be due to the patient’s immunosuppression, which may have caused the cerebral toxoplasmosis lesions to be relatively low in metabolism, resulting in less FDG uptake or no significant increase in FDG, as compared to more virulent bacterial infection. Furthermore, compared to normal tissues, malignant tumors show progressively increasing metabolic activity over time, while the metabolic activity of normal tissues either stabilizes or decreases, resulting in high contrast between malignant lesions and the background [39]. Therefore, the low FDG activity may be a reliable tool for differentiating malignant brain tumors such as PCNSL from cerebral toxoplasmosis, as suggested by a previous report [37]. Coincidentally, the brain MRI findings of Patient 3 exhibited typical imaging characteristics of cerebral toxoplasmosis, and the FDG PET/CT findings also suggested an infectious lesion with less FDG activity than bacterial infection, or a malignant brain tumor such as PCNSL. Therefore, FDG PET/CT brain imaging may add value in the differential diagnosis when patients lack the typical CT or MRI findings for cerebral toxoplasmosis, or when neuroimaging features are overlapped with malignant brain tumor such as PCNSL.

There are a few neuroimaging features associated with cerebral toxoplasmosis, such as the ring enhancement and eccentric or central nuclear target-like enhancement feature, which corresponds to neuropathological features of cerebral toxoplasmosis. A study by Kumar et al. [33] suggested that the typical central enhancement core of cerebral toxoplasmosis was generated by a bundle of inflamed vessels extending downward along the sulcus, surrounded by concentric necrotic zones and a wall composed of tissue cells and proliferating vessels with increased permeability, leading to the peripheral ring-enhancing pattern.

In this report, mNGS testing was performed on blood and/or CSF samples for our case series and for six patients identified from our literature review. Luo et al. [7] and Zhou et al. [28] have reported the usefulness of mNGS for toxoplasmosis diagnosis. Although PCR testing for toxoplasma infection is commonly used in clinical practice, it is still limited by its detection range and sensitivity [40]. Researchers have pointed out that mNGS, as a comprehensive direct detection method, may complement or eventually replace clinical microbiological methods such as culture, antigen detection, and PCR [41]. Although mNGS may not replace traditional diagnostic methods immediately [9], it serves as an adjunct tool for diagnosis of cerebral toxoplasmosis. It may be particularly useful in patients lacking typical neuroimaging findings and when brain biopsies are not feasible.

Currently, the treatment for toxoplasmosis targets acute and reactivated infections caused by the pathogen. As there are no drugs capable of eradicating toxoplasma tissue cysts, the persistence of the parasite in its cystic stage remains a significant obstacle to the effective elimination of toxoplasmosis [42]. If treatment is delayed, mortality in immunocompromised individuals could be high. For Patient 3 in our series, despite five months of intermittent azithromycin and sulfamethoxazole, the patient relapsed after discontinuation of the medication for one month. This patient in our case series had a CD4^+^ T-cell count below 200 cells/ul, indicating severely compromised immune function, which may have been the reason for the recurrence of cerebral toxoplasmosis.

Overall, our case series and literature review support neuroimaging features of cerebral toxoplasmosis for early diagnosis of the disease. Furthermore, toxoplasmosis is a fatal complication in patients after allo-HSCT, especially in children with severe β-TM. Therefore, it is essential to take the necessary preventive measures, including serological screening for toxoplasma antibodies in both recipients and donors prior to transplantation. In patients of positive serological results, a comprehensive prognostic evaluation, personalized treatment plans and prophylactic medications prior to allo-HSCT are recommended. Once the diagnosis is confirmed, prompt treatment is essential to improve survival and prevent relapse. For allo-HSCT patients presenting with unexplained fever, headache, and neurological symptoms in the absence of typical neuroimaging findings, additional diagnostic tests such as blood and CSF mNGS may be helpful to promptly identify and treat toxoplasmosis to prevent adverse outcomes. This study was limited by a small cohort, for which we could not ascertain differential distribution of brain lesions. We could not perform further sub-analysis such as separating the lesions by size. In addition, as a retrospective case series, there were inherent limitations with confounding variables such neuroimaging scan parameters, imaging time points, lack of advanced imaging data such as brain perfusion, diffusion-tensor imaging, and brain functional sequences. Nevertheless, this study should motivate further research on the pediatric population undergoing allo HSCT for severe β-TM.

## Conclusion

The neuroimaging features of cerebral toxoplasmosis, such as ring-like enhancement and eccentric or central nuclear target-like enhancement, may aid in early diagnosis and prompt treatment in children with severe β-thalassemia after allo-HSCT.

## Supplemental data

Supplementary data can be found at the following link: https://www.bjbms.org/ojs/index.php/bjbms/article/view/10708/3407
